# ATP hydrolysis assists phosphate release and promotes reaction ordering in F_1_-ATPase

**DOI:** 10.1038/ncomms10223

**Published:** 2015-12-17

**Authors:** Chun-Biu Li, Hiroshi Ueno, Rikiya Watanabe, Hiroyuki Noji, Tamiki Komatsuzaki

**Affiliations:** 1 Research Institute for Electronic Science, Hokkaido University, Sapporo 001-0020, Japan; 2 Department of Applied Chemistry, Graduate School of Engineering, University of Tokyo, Tokyo 113-8656, Japan; 3 PRESTO, Japan Science and Technology Agency, Tokyo 113-8656, Japan; 4 CREST, Japan Science and Technology Agency, Tokyo 113-8656, Japan

## Abstract

F_1_-ATPase (F_1_) is a rotary motor protein that can efficiently convert chemical energy to mechanical work of rotation via fine coordination of its conformational motions and reaction sequences. Compared with reactant binding and product release, the ATP hydrolysis has relatively little contributions to the torque and chemical energy generation. To scrutinize possible roles of ATP hydrolysis, we investigate the detailed statistics of the catalytic dwells from high-speed single wild-type F_1_ observations. Here we report a small rotation during the catalytic dwell triggered by the ATP hydrolysis that is indiscernible in previous studies. Moreover, we find in freely rotating F_1_ that ATP hydrolysis is followed by the release of inorganic phosphate with low synthesis rates. Finally, we propose functional roles of the ATP hydrolysis as a key to kinetically unlock the subsequent phosphate release and promote the correct reaction ordering.

## Abstract (web summary)

F_1_-ATPase is a rotary motor protein that can efficiently convert chemical energy of ATP hydrolysis to mechanical work. Here, the authors study its catalytic reactions using high-speed single-molecule observations and contemporary time series analysis, and propose a lock and key type mechanism.

## Graphical abstract

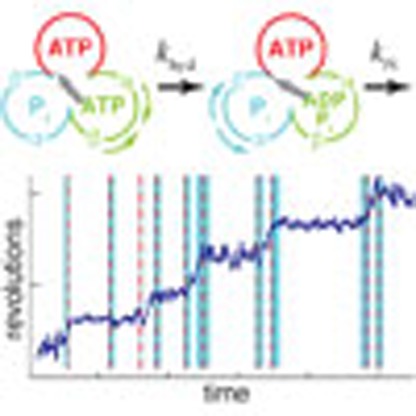

The rotary protein motor F_1_-ATPase (F_1_) with subunits 
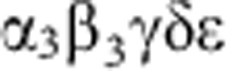
 is a catalytic sub-complex of the F_o_F_1_-ATP synthase that catalyses the synthesis of ATP from ADP and inorganic phosphate (P_i_)^1^,^2^,^3^. When operating in reverse direction, F_1_ hydrolyzes ATP to rotate the rotor γ-subunit against the hexameric ring-shaped stator α_3_β_3_. The sub-complex α_3_β_3_γ is the minimum component for F_1_ to operate. F_1_ has three catalytic sites located at the αβ interfaces and ɛ hosted mainly by the β-subunits. The rotation of the γ-subunit is induced by the transitions among catalytic states and cooperative conformation changes of the three β-subunits^4^,^5^. Precise torque experiments^6^,^7^,^8^ have shown that F_1_ is a highly efficient motor that can convert almost all of the chemical free energy of hydrolysis to the mechanical energy of rotation.

To understand the working principles and the fine coordination of the conformation changes of the β-subunits, extensive studies have been carried out to unveil the tight chemomechanical couplings between the rotation and the catalytic states as shown in Fig. 1 for F_1_ derived from thermophilic *Bacillus* PS3. F_1_ performs counterclockwise step-like rotation (viewed from the membrane side) of 120° steps each coupled with the hydrolysis of a single ATP^6^. The 120° step further decomposes into 80° and 40° substeps^9^ (85° and 35° substeps for *Escherichia coli* F_1_-ATPase^10^). It was found that the 80° substep is triggered by ATP binding and ADP release^9^,^11^,^12^ at different β-subunits, while the 40° substep is induced by ATP hydrolysis and P_i_-release^12^,^13^,^14^ that also occur at different β-subunits^15^,^16^ (see Fig. 1). The dwells before the 80° and the 40° substeps are termed the binding and the catalytic dwells, respectively.

On the other hand, single F_1_ stall-and-release experiment^17^ shows that the catalytic rates of F_1_ are modulated by the rotary angle of the γ-subunit. Specifically, the rate constants of ATP binding and P_i_-release are found to be highly dependent on the γ-angle, suggesting that these two reactions are the major torque-generating steps^17^,^18^. In contrast, the ATP hydrolysis is less affected by the γ rotation and, therefore, does not contribute much to the torque-generation. Furthermore, quantum mechanical/molecular mechanical calculations^19^ demonstrate that the chemical free energy released from hydrolyzing a bound ATP at the F_1_ catalytic site is ∼10 pN·nm, which is relatively small compared with the free-energy Δ*G* released from hydrolyzing one ATP in a solution^7^,^20^, for example, Δ*G*∼−90 pN·nm under physiological conditions: [ATP] ∼1 mM, [ADP] ∼0.1 mM and [P_i_] ∼1 mM. With its insignificant contributions to the torque-generation and the overall chemical energy released, it remains elusive what functional role the hydrolysis of the bound ATP can play in the F_1_ catalytic cycle.

In this study, we investigate the detailed kinetics of the catalytic dwells and scrutinize the possible roles of the ATP hydrolysis reaction in terms of single F_1_ rotary observations with microsecond time resolutions and contemporary time series analysis. In particular, model-free change point (CP) and clustering analyses are applied to the angular traces from free rotations of wild-type (WT) F_1_ derived from thermophilic *Bacillus* PS3 to robustly construct the statistics of waiting time and angular fluctuations of the catalytic dwells with short duration ∼1 ms. This allows us to detect a small angular increment during the catalytic dwell triggered by the ATP hydrolysis reaction that is indiscernible in previous studies using conventional analysis methods. Moreover, we find in freely rotating F_1_ that ATP hydrolysis is followed by P_i_-release with low synthesis rate compared with the hydrolysis rate. We then propose the functional roles of ATP hydrolysis as a key to accelerate (or kinetically unlock) the subsequent P_i_-release reaction and promote the correct reaction ordering, despite its minor contributions to the torque and chemical energy generations.

## Results

### Observing the rotary motions of single F_1_-ATPase

WT F_1_ derived from thermophilic *Bacillus* PS3 are prepared as described in ref. 21. To monitor the rotary motions of F_1_, the stator complex (α_3_β_3_) is fixed to a glass surface and a colloidal gold bead as a rotation probe with diameters of 40–80 nm is attached to the rotor γ-subunit^15^. The freely rotating beads were then observed at room temperature under a custom-built laser dark-field microscope^22^. To focus on the catalytic dwells, the rotation assay was performed at high ATP concentration ([ATP]=4 mM), such that the binding dwells become extremely short (∼10 μs) and are negligible. With high image recording rates of 27,000–100,000 frames per s, typical rotary traces show clear pause and rotation regions. An example is shown in Fig. 2a.

### Identifying catalytic dwells by CP and clustering analyses

To obtain the catalytic dwell statistics reliably from the rotary trace free from artefacts that may arise from common analysis methods (for example, thresholding and binning)^23^,^24^,^25^, CP-detection scheme based on permutation test^26^ without the need to assume *a priori* noise model is generalized to locate the time instants where changes in the linear trends occur. An example of outcomes from the CP detection applied to the rotary trace is illustrated in Fig. 2a. The detection includes statistically testing the existence of CPs, and determining the most probable CP locations with uncertainty estimation (detailed in Methods and Supplementary Notes 1–3, and validations in Supplementary Fig. 6). The uncertainties of CP locations are essential to estimate the corresponding errors in the catalytic dwell statistics. It is evident from Fig. 2a that the detection scheme can automatically identify CPs separating the pause and rotation segments in the time series.

Because of some undesired fluctuations in the measurements, extra CPs caused by actual bead motions may exist (for example, the CP indicated by the arrow in Fig. 2a). Therefore, CP detection is followed by a clustering procedure to classify the detected CP intervals into pause and rotation intervals, and to assign the pause intervals accordingly to the three different catalytic dwells separated by 120°. In this study, we employ an information-based soft clustering method^27^,^28^ (detailed in Methods and Supplementary Note 4, and validations in Supplementary Figs 6,7) to assign each CP intervals to the three different catalytic dwells with the conditional probability, *P*(*C*_*α*_|*e*_*i*_), for a given CP interval *e*_*i*_ (*i*=1, …, *N*_*e*_ with *N*_*e*_=total number of detected CP intervals) to belong to the catalytic dwell *C*_*α*_ (*α*=1, 2 and 3). The clustering results are illustrated in the stacked bar chart in Fig. 2a with red, green and cyan bars labelling the conditional probabilities for a given CP interval to be assigned to the three catalytic dwells. Next, a CP interval is identified as a pause interval if it can be assigned to any catalytic dwell with >95% probability, and is identified as rotation intervals otherwise. This corresponds to a possible 5% assignment error for the pause intervals. Finally, extra CPs between consecutive pause intervals (for example, indicated by the arrow in Fig. 2a) assigned to the same catalytic dwell are removed.

### Dwell-time statistics

The CP detection and clustering procedure results in a set of catalytic dwells from which various statistics can be extracted. Figure 2b shows the dwell-time survival probabilities for each of the three catalytic dwells from a single F_1_ attached to a 40 nm bead. In contrast to dwell-time histograms commonly used, survival probabilities are constructed in our analysis, since they do not require to introduce artificial binnings. The survival probabilities are well fitted by double exponential curves (*c*_1_ exp(−*t*/*τ*_1_)+*c*_2_ exp(−*t*/*τ*_2_)) with two distinct time constants *τ*_1_∼0.2 ms and *τ*_2_∼1 ms, which agrees with previous studies^13^ that there exist two catalytic processes (ATP hydrolysis and P_i_-release) at the catalytic dwells. As can be seen from Fig. 2b, the observed time constants from different catalytic dwells of the same molecule vary, which can be caused by the heterogeneity of local environment around the motor. Therefore, dwell-times from different catalytic dwells are not mixed together in our analysis to avoid introducing spurious results. In addition, correlations between dwell times of successive catalytic pauses are investigated in terms of two-dimensional (2D) correlograms (Supplementary Fig. 9) and no apparent correlation can be detected.

**Figure 1:**
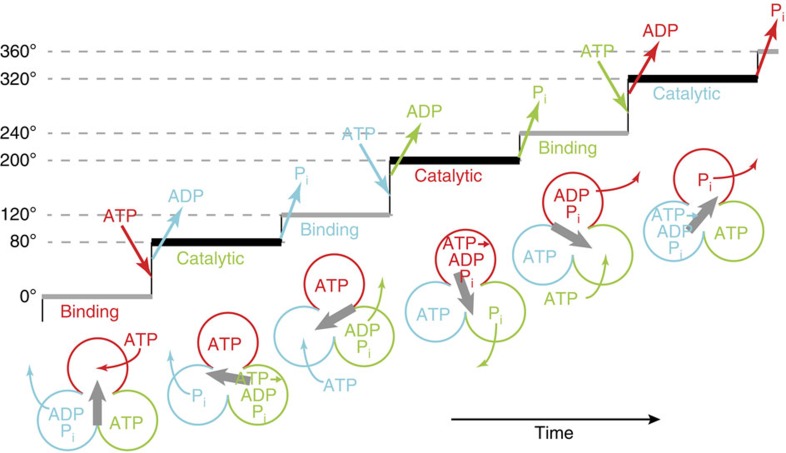
Coupling between catalysis and rotation in F_1_. Dwells in the γ rotation and the corresponding nucleotide states at each β-subunit (coloured circles) are shown. Nucleotides are coloured according to the β-subunits for visual clarity. The grey arrows represent the rotary angles of the γ-subunit. ATP binding, hydrolysis, ADP release and P_i_ release of the red β-subunit occur at 0°, 200°, 240° and 320°, respectively.

**Figure 2:**
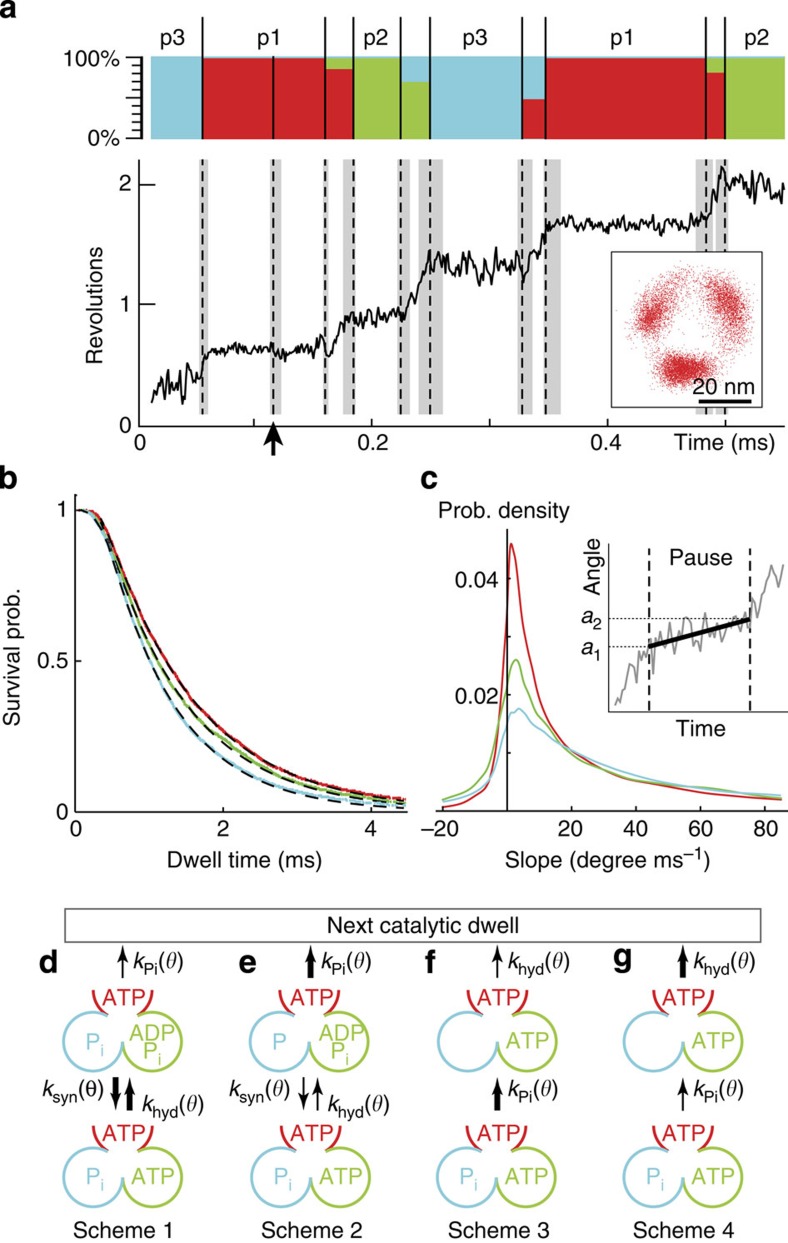
Catalytic dwell statistics from CP and clustering analyses. (**a**) The rotary trace (solid line) and the detected CPs (dash lines). Grey regions: uncertainties in CP locations. Inset: a typical rotary trace on the camera *x*–*y* plane. The arrow indicates an extra CP due to undesired fluctuations that will be removed by the subsequent clustering. The stacked bar chart shows results from soft clustering. Ordinate: conditional probabilities of a given CP interval assigned to the first (red), second (green) and third (cyan) catalytic dwells. CP intervals that can be assigned to any catalytic dwell with probability ≥95% are denoted as pauses (pause 1 (p1), pause 2 (p2) or pause 3 (p3)). CPs between two pause intervals of the same catalytic dwells are then removed. (**b**) Dwell-time survival probabilities (dash lines) of the three catalytic dwells from a single F_1_ (for 40 nm bead case). Colour lines: double exponential fits with time constants *τ*_1_∼0.2 ms and *τ*_2_∼1 ms. The colour scheme is the same as in **a**. (**c**) Slope distribution of the three catalytic dwells for the single F_1_ as **b**. The slope is obtained by fitting the pause interval with linear trend (inset). *a*_1_ and *a*_2_: initial and final angle of the fitted line. (**d**–**g**) Four possible scenarios for the catalytic dwells with angle-dependent rate constants. Nucleotide states of the dwell at 80° in Fig. 1 are shown for illustration. Thicker (thinner) arrows represent larger (smaller) rate constants.

To take into account the two observed time constants and the lack of correlations, we consider the four simplest scenarios for the catalytic dwell as shown in Fig. 2d–g, where the chemical states of the catalytic dwell at 80° in Fig. 1 is depicted as illustration. Specifically, the P_i_-release (at cyan β-subunit) follows the hydrolysis (at green β-subunit) in Fig. 2d–e, and vice versa in Fig. 2f–g. These imply that the power stroke connecting the current dwell with the next is triggered by the P_i_-release in Fig. 2d,e and by the hydrolysis in Fig. 2f,g, respectively. Moreover, the P_i_-release is rate-limiting in Fig. 2d,g, and the hydrolysis/synthesis are rate-limiting in Fig. 2e,f. The rate constants, denoted by *k*_hyd_(*θ*), *k*_syn_(*θ*) and *k*_Pi_(*θ*) for hydrolysis, synthesis and P_i_-release, respectively, are in general γ-angle (*θ*) dependent^17^. In these schemes, the P_i_ binding is ignored because of the low P_i_ concentration considered in the current study. On the other hand, the synthesis reactions are neglected in Fig. 2f,g, where the power stroke is assumed to be triggered by the hydrolysis, implying that the chance of backward reaction (rotation) is low. In the following, we will show that our analyses of the angular fluctuations and their effects on the catalytic kinetics support the scenario in Fig. 2e with low synthesis rates.

### Small rotation at the catalytic dwell

We first look at the slope distribution of the fitted linear trend of the pause intervals (Fig. 2c). The significant bias of distributions to the positive slopes indicates that on average there exists a small angular increment during the catalytic dwell. We propose that this angular increment originates from a small angular shift in the equilibrium angles (or free-energy minima) of the two catalytic states (for example, from the pre- to post-hydrolysis states in Fig. 2e) at the catalytic dwell. Although this angular increment is evident from the positive skewness of slope distribution, it is, however, too small to be resolved from angular histograms of the catalytic dwells considered in previous studies (Supplementary Fig. 8). We also note that the application of CP and clustering analyses to separate the rotation from the pause intervals is crucial in obtaining the correct slope distribution of the catalytic dwells free from false contribution from the rotation parts of the trace (see validations in Supplementary Figs 6,7). On the other hand, an angular shift after hydrolysis or P_i_-release is not uncommon in F_1_ motors. It was reported recently in human mitochondrial F_1_ (ref. 29) that the hydrolysis and P_i_-release occur at separated dwells and trigger γ rotations of ∼30° and ∼25°, respectively.

### Modelling the kinetics of the catalytic dwell

**Figure 3:**
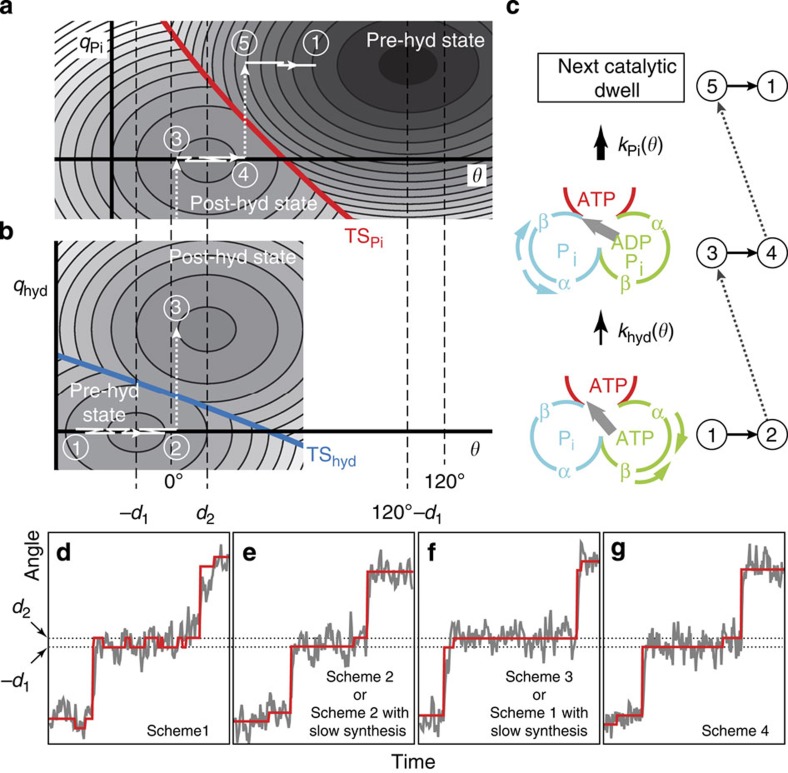
Free-energy surfaces for the catalytic dwell and typical simulation traces. (**a**–**b**) 2D P_i_-release (**a**) and hydrolysis (**b**) reaction diagrams for the scheme in Fig. 2e with slow synthesis (not to scale). *q*_hyd_ and *q*_Pi_ are reactive coordinates. The post-hydrolysis state on the product side for the hydrolysis is redrawn on the reactant side for the P_i_-release. Free-energy surfaces are represented by equi-energy contours with darker colours for lower energies. The reactant and product surfaces intersect at TS_hyd_ and TS_Pi_. The catalytic dwells locate around *θ*=0° and 120°, and the equilibrium angles of the pre- and post-hydrolysis states locate at *θ*=−*d*_1_ and *d*_2_, respectively. A schematic representation of reaction–diffusion path during one catalytic dwell is shown with numberings by white solid (diffusion) and dash (reaction) lines (**1**→**2** and **3**→**4**: diffusion along *θ*; **2**→**3** and **4**→**5**: hydrolysis and P_i_-release reaction; **5**→**1**: power stroke). For visual clarity, small offsets are introduced in the white solid lines for the diffusion along *θ*. (**c**) Correspondence between the chemical states and the numbered trajectory in **a**,**b**. The relative orderings of the α- and β-subunits and the associated conformational changes at the catalytic dwell (see Discussion for details) are also indicated. (**d**–**g**) Typical simulated traces (for 40 nm bead case) of different schemes (with synthesis rates comparable to or much lower than the hydrolysis rates) in Fig. 2d–g. Subtitles indicate the corresponding scheme(s) for the traces. Grey lines: simulated rotary trace. Red lines: equilibrium angle of the states where the system resides.

To integrate two catalytic states, the rate constants modulated by the γ-angle and the small angular increment at the catalytic dwell, we adopt a reaction–diffusion framework^18^,^30^ originally proposed in the study of electron transfer reaction under the effects of solvent orientation motions^31^. In this framework, the F_1_ catalytic kinetics is modelled by 2D reaction diagrams to account for the chemomechanical couplings between the catalytic reactions and rotary fluctuations. As a schematic illustration, the model is shown in Fig. 3a,b for the scheme in Fig. 2e with low synthesis rates (relative to hydrolysis). For each reaction (hydrolysis and P_i_-release), the reaction diagram is characterized by two coordinates: the mechanical coordinate (γ-angle *θ*) and a 1D reactive coordinate (*q*_hyd_ and *q*_Pi_). The two reaction diagrams for hydrolysis and P_i_-release are depicted separately since *q*_hyd_ and *q*_Pi_ are generally different. The free-energy surfaces of the catalytic states (the pre- and post-hydrolysis states), which are assumed to be harmonic in *θ*, are represented by the equi-energy contours and intersect at the transition states (TS_hyd_ and TS_Pi_) separating the reactant and product. The relaxations of *q*_hyd_ and *q*_Pi_ are assumed to be fast enough compared with the timescales of reactions and rotary fluctuations such that equilibrium in the reactive coordinate is always attained on the reactant's and product's surfaces.

A natural consequence from the inclusion of the mechanical coordinate *θ* in the reaction diagrams is that the activation energy depends on the γ-angle, giving rise to the angle-dependent rate constants^31^. Moreover, we introduce an angular difference in the free energy minimum of the pre- and post-hydrolysis states (located at *θ*=−*d*_1_ and *d*_2_ in Fig. 3a,b) to account for the small angular increment during the catalytic dwell. In the example of Fig. 3a,b, the γ-angle diffuses under the influence of the free-energy surface in the reactant that describes the rotary potential exerted by the α_3_β_3_ rings to the γ-subunit. During the diffusion along *θ*, the system can react from the reactant to the product state with rate constants *k*_hyd_(*θ*) and *k*_Pi_(*θ*). We perform simulations of the reaction–diffusion dynamics of γ-angle on the free-energy surfaces in terms of the overdamped Langevin dynamics and Monte Carlo techniques, where the model parameters of reactions (*k*_hyd_(*θ*), *k*_syn_(*θ*) and *k*_Pi_(*θ*)) and diffusion (relaxation time, angular increment and potential width) are extracted from the experimental data (detailed in Methods).

Following previous studies^17^, we adopt an exponential angle dependence for the rate constants of hydrolysis and P_i_-release, *k*_hyd_(*θ*)∝exp(*b*_hyd_*θ*) and *k*_Pi_(*θ*)∝exp(*b*_Pi_*θ*) with *b*_hyd_∼0.02 degree^−1^ and *b*_Pi_∼0.12 degree^−1^, and an angular independent *k*_syn_(*θ*)=*k*_syn_. We note that *k*_Pi_(*θ*) has the strongest *θ* dependence (increases more than 10 times when *θ* increases 20°), implying that the P_i_-release reaction is significantly decelerated when the viscous drag of the probe increases (that is, when the relaxation time of rotary fluctuations increases) as demonstrated recently by Watanabe *et al.*^18^. The hydrolysis and synthesis reactions, on the other hand, are insensitive to the viscous drag of the probe because of the weak *θ* dependence of *k*_hyd_(*θ*) and *k*_syn_.

The reaction diagrams for the other schemes in Fig. 2d–g are similar to the one in Fig. 3a,b. For example, for Scheme 4 of Fig. 2g, the reactant and product states in Fig. 3b are replaced with the pre- and post-P_i_-release states with the corresponding reactive coordinates and TSs swapped between Fig. 3a,b.

A schematic representation of the reaction–diffusion processes and the corresponding nucleotide states at the catalytic dwell is shown in Fig. 3a–c. Starting at step **1**, the system diffuses in the pre-hydrolysis state during **1**→**2**. Upon ATP hydrolysis (**2**→**3**) at the green β-subunit in Fig. 3c, the system reacts to the post-hydrolysis state, where the system diffuses during **3**→**4**. Steps **1**–**4** correspond to the catalytic pause interval identified by CP detection for the experimental rotary traces, and a small angular increment (Δ*θ*=*d*_2_+*d*_1_) is introduced upon the reaction **2**→**3**. The current dwell ends at step **4** when P_i_-release occurs (**4**→**5**) to bring the system to the pre-hydrolysis state of the next catalytic dwell (near 120°). After landing at step **5** locating at the high-energy region of the potential, a power stroke (**5**→**1**) takes place and the system relaxes to around the potential minimum. The next catalytic dwell starts again at step **1** and the above process repeats.

### Determining the rate-limiting step at the catalytic dwell

Rotary traces mimicking the 40 nm bead case are simulated for different schemes in Fig. 2d–g. Typical traces are shown in Fig. 3d–g and the equilibrium angles of the states where the system resides are indicated by red lines. It is evident from the traces of the equilibrium angle that the nature of angular fluctuations at the catalytic dwells varies with the schemes. As there are two distinct time constants (∼0.2 and 1 ms) obtained by experiments at the catalytic dwell for the case of 40 nm bead, there are three distinct types of angular fluctuations: (1) the equilibrium angle frequently switches between the minima at *θ*=−*d*_1_ and *d*_2_ during the catalytic dwell (Fig. 3d). This corresponds to Scheme 1 where the system can make frequent transitions between the pre- and post-hydrolysis states because of slow P_i_-release; (2) the equilibrium angle stays long at *θ*=−*d*_1_ and then briefly at *θ*=*d*_2_ (Fig. 3e,g). This corresponds to the cases where the reverse reaction rarely occurs and the first process at the catalytic dwell is rate-limiting; and (3) the equilibrium angle stays shortly at *θ*=−*d*_1_ but for a longer time at *θ*=*d*_2_ (Fig. 3f). This corresponds to the cases where the reverse reaction rarely occurs and the second process at the catalytic dwell is rate-limiting.

**Figure 4:**
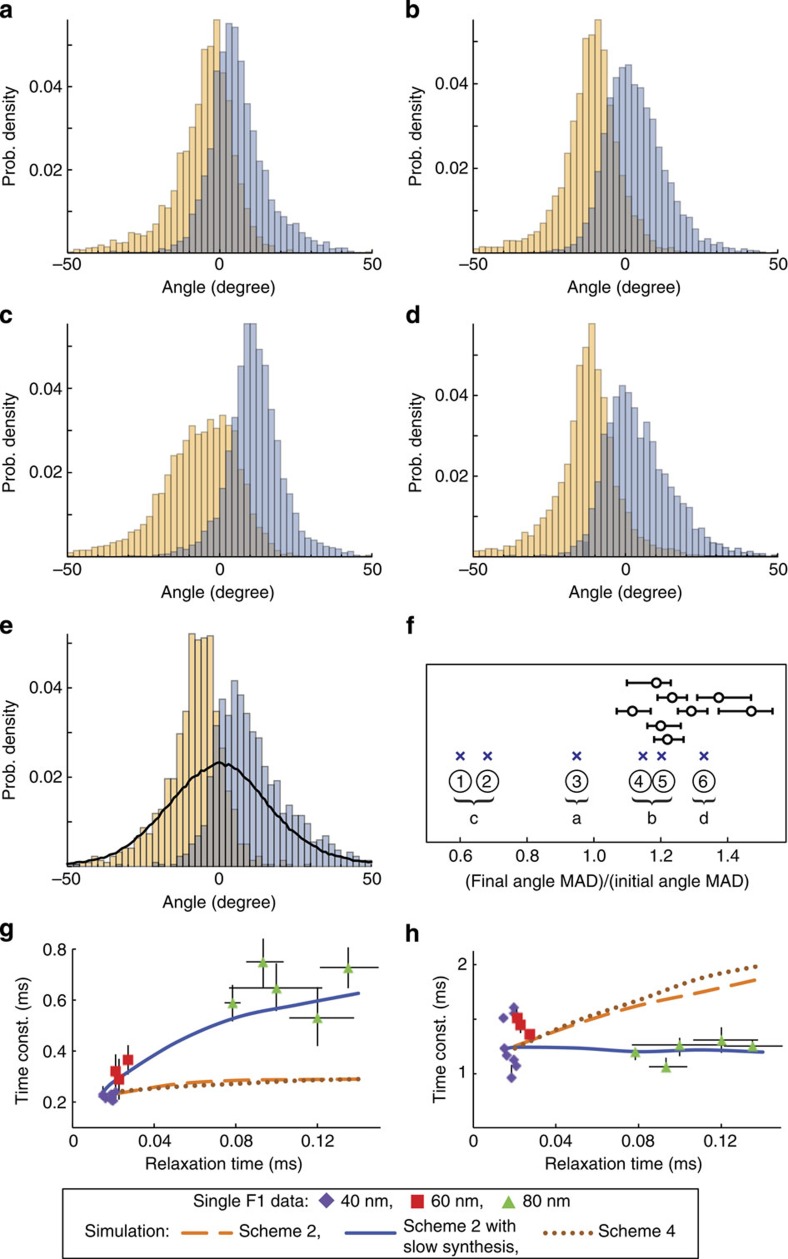
Initial and final angle distributions and relaxation time dependence of catalytic time constants. (**a**–**d**) Initial- (yellow) and final-angle (grey) distributions for the four simulation cases (with 40 nm bead) in Fig. 3d–g. (**e**) Typical initial- and final-angle distributions from one catalytic dwell of a single F_1_ (with 40 nm bead). Solid line: angle distribution of the catalytic dwell. (**f**) Ratios of the final- and initial-angle MAD from experimental data with 40 nm bead (circles) and simulations (crosses). Numbering of crosses: (1) Scheme 3; (2) Scheme 1 with slow synthesis; (3) Scheme 1; (4) Scheme 2; (5) Scheme 2 with slow synthesis; and (6) Scheme 4. The corresponding initial- and final-angle distributions are also indicated. Error bars represent the 68% confidence intervals of the MAD ratio. (**g**,**h**) Catalytic time constants versus relaxation time of angular fluctuations from experiments and simulations. Smaller time constant (**g**) and for the Larger one (**h**). Error bars: uncertainties in the estimated relaxation times and time constants fitted from rotary traces. Each point in **f**–**h** corresponds to one catalytic dwell from a single F_1_.

Compared with the experimental data, these differences in the angular fluctuations provide useful clue in identifying the correct scheme for the catalytic dwell. In particular, we consider the statistics of initial- and final-angle of the linear trend of the pause intervals (*a*_1_ and *a*_2_ defined in Fig. 2c) to differentiate the above three types of angular fluctuations. The initial- and final-angle distributions for the four simulation cases in Fig. 3d–g are shown, respectively, in Fig. 4a–d, and they can be distinguished by the widths of their initial- and final-angle distributions. To quantify the degree of statistical dispersion, we evaluate the median absolute deviation (MAD) that is less influenced by outliers and skewness of the distribution^32^. For a list of values {*x*_1_, *x*_2_, ⋯, *x*_*n*_} with median 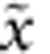, MAD is the median of the list 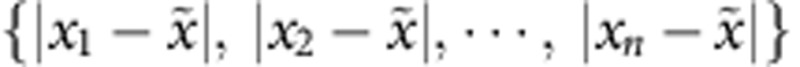. In Fig. 4f, we compare the ratios of final- and initial-angle MADs between the experimental data with 40 nm bead and those from simulations. It is clear that the experimental data favours the initial- and final-distributions given in Fig. 4b,d, that is, the second type of angular fluctuations in Fig. 3e,g corresponding to Scheme 2, Scheme 2 with slow synthesis and Scheme 4. This result suggests that the rate-limiting step is the first catalytic process at the catalytic dwell. Moreover, in order for the traces in Fig. 3e,g to produce a ratio of final- and initial-angle MADs matching the experimental ones (∼1.2), an angular increment Δ*θ*=*d*_2_+*d*_1_∼20° is required (see Methods). It remains to determine which process (hydrolysis or P_i_-release) proceeds first at the catalytic dwell.

### Hydrolysis followed by P_i_-release with low synthesis rates

Since the angular fluctuations in Fig. 3e,g are quite similar for the case of 40 nm bead, other features of the reaction–diffusion systems are needed to determine the ordering of hydrolysis and P_i_-release. Here we use the strong sensitivity of P_i_-release reaction to the relaxation time of angular fluctuations for the identification. By performing single F_1_ rotary measurements with various bead sizes (40, 60 and 80 nm) under the same experimental conditions, we monitor the changes of the two observed time constants at the catalytic dwell as a function of relaxation time as shown in Fig. 4g,h, where the relaxation times of the experimental rotary traces are determined by evaluating the autocorrelation of angular fluctuation at the catalytic dwells. Figure 4g–h show that the smaller time constant from the single F_1_ data is sensitive to the relaxation time, whereas the larger one does not. In the same figure, we also show the dependence of time constants on the relaxation time from simulations. Only Scheme 2 with slow synthesis compared with the hydrolysis can explain the strong sensitivity of the smaller time constant on the relaxation time, since in that case the smaller time constant corresponds to the faster P_i_-release reaction (Fig. 2e). In contrast, the smaller time constant from Scheme 4 corresponds to the hydrolysis reaction (Fig. 2g), which is insensitive to the relaxation time.

The situation is more subtle in the case of Scheme 2. Since Scheme 2 contains the synthesis (backward) reaction (Fig. 2e), the smaller time constant roughly characterizes reaction paths that do not involve the backward reaction, that is, time constant for hydrolysis directly followed by P_i_-release, and the larger time constant corresponds to reaction paths that involve several hydrolysis and synthesis reactions (Fig. 2e). As the relaxation time of angular fluctuation increases, the P_i_-release reaction decelerates significantly which causes an increase in the number of cycles of hydrolysis and synthesis reactions, resulting in the strong sensitivity of the larger time constant on the relaxation time.

Our result, that P_i_-release is the process just before the power stroke, is consistent with the expectation^7^,^15^ that P_i_-release, instead of the hydrolysis, is the major torque-generation step at the catalytic dwell attributed to the strong angular dependence of *k*_Pi_(*θ*).

### Role of hydrolysis as a key to accelerate P_i_-release

Figure 3a–c provide a summary of the above results in terms of the 2D reaction diagrams. With a strong angular dependence of *k*_Pi_(*θ*), it can be seen from the figure that the angular increment from pre- to post-hydrolysis states brings the system towards the lower activation barrier regions of the P_i_-release TS_Pi_ to react. The angular increment, Δ*θ*=*d*_1_+*d*_2_∼20°, accelerates the P_i_-release reaction more than 10 times as if the system would cross the P_i_-release activation barrier at the pre-hydrolysis state, that is, at *θ*∼−*d*_1_. We therefore propose a functional role of hydrolyzing the bound ATP as a ‘key' to accelerate (or kinetically ‘unlock') the subsequent P_i_-release reaction to complete the catalytic cycle, even though the hydrolysis process is not a major torque-generating step and the chemical free energy produced is notably small compared with those from the ATP binding and P_i_-release reactions.

Another implication from the reaction diagrams in Fig. 3a,b and the role of hydrolysis as a key is the promotion of correct reaction ordering at the catalytic dwell, that is, the P_i_-release (the torque-generating step) should follow the hydrolysis to avoid rotating away from the catalytic dwell before the hydrolysis of ATP occurs. More precisely, suppose the system is at the pre-hydrolysis state with *θ*∼−*d*_1_ and it can proceed either with the hydrolysis (at the green β-subunit) or P_i_-release (at the cyan β-subunit) by crossing the TS_hyd_ or TS_Pi_ (Fig. 3a–c). A simple estimation using the angle dependence of the rate constants (see Methods) shows that *k*_hyd_(*θ*=−*d*_1_) is more than 10 times larger than *k*_Pi_(*θ*=−*d*_1_). This implies that it is unlikely to have the P_i_-release and power stroke occurring before the hydrolysis (that is, turning the key).

## Discussion

By extracting reliable dwell statistics from high-speed single-molecule imagings in terms of contemporary CP and clustering analyses, the aims of the present study are to reveal the detailed kinetics and the possible roles of bound ATP hydrolysis at the catalytic dwells of WT F_1_ free from any applied stalling force. To establish a connection with the conformational changes of the α- and β-subunits, we correlate our results with recent conformational studies of F_1_ in the following. It was found^16^,^33^,^34^ that the principal conformation changes at the α_3_β_3_ stator ring during the catalytic cycle involve the close/open transitions of the β-subunits upon nucleotide bindings/release, and the tighten/loosen transitions of the αβ interfaces upon hydrolysis and P_i_-release. In particular, structural comparison of F_1_ crystal structures^16^ and molecular dynamics simulation^34^ show that at the catalytic dwell shown in Fig. 3c, the αβ interface (green α and β) with ATP bound at 200° before becomes tighter to facilitate hydrolysis. We expect that this prominent tightening motion can only induce a small γ-angle increment (∼20° suggested by our analysis) from pre- to post-hydrolysis states (Fig. 3c), probably due to the weak interaction between the β- and γ-subunits during the hydrolysis reaction, as implied by the weak γ-angle dependence of *k*_hyd_(*θ*) (ref. 17) and the recent finding^35^ that the major contact region at the β-subunit (a conserved DELSEED helical loop located at the C-terminal domain of the β-subunit) with the γ-subunit does not contribute much to the torque transmission associated with the ATP hydrolysis reaction.

On the other hand, structural and conformational studies^36^,^37^,^38^ also suggest that at the catalytic dwell the β-subunit which bound ATP at 200° before (green β in Fig. 3c) strongly interacts with the two neighbouring α-subunits (green and cyan α's in Fig. 3c) such that motions of the two αβ interfaces (green and cyan) well correlate with each other. In particular, the tightening of the green αβ interface for hydrolysis is expected to trigger the loosening of the cyan αβ interface in Fig. 3c, resulting in a decrease in the binding affinity of P_i_ (ref. 16) to be released at the end of the catalytic dwell. We therefore propose that the role of bound ATP hydrolysis as a key to assist the release of P_i_ may correspond to this tightly correlated αβ interface motions. Similarly, without the occurrence of the hydrolysis reaction, the αβ interface with the bound P_i_ (cyan αβ in Fig. 3c) would remain tight and thus, it is unlikely for the P_i_-release to happen. Furthermore, the close interplay between couplings among different subunits in the α_3_β_3_ stator rings and the fine coordination of reaction sequence does not occur only at the catalytic dwell. It was observed using high-speed atomic force microscopy^38^,^39^ that the α_3_β_3_ stator ring still undergoes cyclic conformation changes even without the rotor γ-subunit, in which the open-to-close transition of the β-subunit induced by the ATP binding also behaves like a key to expedite the close-to-open transition of an adjacent β-subunit for the release of ADP. We therefore expect that such unlock mechanism could be a common strategy to achieve precise chemomechanical coordination in the F_1_. It was reported^29^, however, in human mitochondrial F_1_ that P_i_-release (at cyan β in Fig. 3c) precedes hydrolysis (at green β in Fig. 3c). In that case, the release of P_i_ may instead serve as the key to expedite the ATP hydrolysis.

Finally, we point out that the slow synthesis reaction at the catalytic dwell suggested here from free rotation is different from previous single F_1_ observations with applied force to stall the rotation of the γ-subunit^15^,^17^ for a long enough time, where the rate constant of synthesis are found to be comparable with those of the hydrolysis. We speculate that this difference may originate from some non-equilibrium features of the freely rotating WT F_1_ at the short (∼ms) catalytic dwell. A precise estimation of the synthesis rates under non-equilibrium conditions will be focused in the future studies.

## Methods

### Data-selection criteria

Rotary traces from measurements are first plotted on the camera *x*–*y* plane to check for circular symmetry. Traces significantly deviated from a circle are not used in the analysis, since the system may be affected by unknown factors such as interaction of the probe with the glass surface, improper fixation, defected molecule and so on. After the above selection, five molecules from the 40 nm bead case, four molecules from the 60 nm case and five molecules from the 80 nm case are considered. The number of pause intervals associated with a single catalytic dwell from any single F_1_ molecule is in general larger than 1,500. On the other hand, catalytic dwells having extremely distinct statistics, that is, outliers, from the majority are not included in analysis.

### Curve fitting

The least squares fitting is employed in our study to fit survival probabilities and autocorrelation functions with exponential functions. The number of exponentials is varied starting from one. The appropriate number of exponentials is then determined as the squared error between the fitted function and the survival probabilities (or autocorrelation function) becomes unchanged as the number of exponentials increases.

### Change point analysis

CP analysis based on permutation test^26^ is generalized to detect changes of linear trend in the rotary traces. A CP is the time instant where the linear trends are different before and after. The major steps in CP detection are as follows.

Firstly, testing the existence of CPs by permutation method. CP detection is treated as a statistical hypothesis test with the two hypotheses: no CP exists versus at least one CP exists. In terms of permutation method, the likelihood that CPs exist in the trace is compared with the likelihood that there is no CP. We declare the existence of CPs if the hypothesis of no CP can be rejected when a given confidence level, for example, 98%, is reached. The method to test the existence of CPs is discussed in detail in the Supplementary Note 1 and Supplementary Figs 1,2.

Secondly, determining the location of change point and its uncertainty. Once the existence of a CP is confirmed, the most probable location of the CP is assigned as the time instant whose left and right segments are best fitted by linear trends in terms of the squared errors. Moreover, the uncertainty in CP location is estimated using the bootstrapping method^40^. The size of the uncertainty depends on both the signal-to-noise ratio of the time series and the number of data points before and after the CP. Since uncertainties in the location of the detected CPs directly affect the dwell-time statistics, we keep track of the error by propagating^41^ the error in the CP location to the dwell time, that is, the time difference between two successive CPs. The set of dwell times and their uncertainties are then subjected to the subsequent fitting to determine the time constants and their uncertainties. The method to determine the CP location and its uncertainty is discussed in detail in the Supplementary Note 2 and Supplementary Fig. 3.

Thirdly, detecting multiple change points recursively. The above steps are repeated with binary segmentation to detect multiple CPs in the rotary trace. The segmentation stops when no new CP can be found with the given confidence level. The method to detect multiple CPs is discussed in detail in the Supplementary Note 3 and Supplementary Fig. 4.

### Clustering to assign CP intervals to catalytic dwells

Here we employ a soft clustering algorithm based on the rate distortion theory in information theory^27^,^28^. The basic idea behind the algorithm is to treat clustering as ‘compressing' the description of data set (the set of CP intervals in our case) into clusters (the three catalytic dwells in our case), while maintaining the distortion to a desired level. Owing to the fact that different catalytic dwells have separated distributions of the rotary angle, the distortion is chosen in our analysis to be the average intra-cluster angle difference in the resulting clusters. Generally, if one compresses the data set, for example, by using a few number of clusters, some details of the data set may be lost, implying the compressed description is ‘distorted' compared with the data set. Therefore, it is always a tug-of-war between compression and maintaining small distortion. A self-contained discussions of the theory, properties and scheme to perform soft clustering of the CP intervals into different catalytic dwells are detailed in the Supplementary Note 4 and Supplementary Fig. 5. The validations of CP and clustering analyses on simulated traces are given in Supplementary Figs 6,7.

### Numerical simulation of the reaction–diffusion model

We simulate the diffusion of the γ-angle *θ* at a reaction state (for example, the pre-hydrolysis state) on the 2D reaction diagram by the overdamped Langevin equation on a harmonic well, *dθ*/*dt*=−*λ*(*θ*−*θ*_e_)+*R*(*t*). Here *θ*_e_ is the equilibrium angle, *λ*=*ω*^2^/*η* (*η*: friction coefficient, *ω*: frequency of the harmonic well) is the drift coefficient characterizing the relaxation time of *θ* (that is, 〈*θ*(*t*)*θ*(*0*)〉∼exp(−*λt*)). *R*(*t*) is a Gaussian random force exerting on *θ* with mean 〈*R*(*t*)〉=0 and correlation 〈*R*(*t*)*R*(*t*′)〉=2*λσ*^2^*δ*(*t*−*t*′), where *σ*^2^ is the variance of the equilibrium angular distribution (that is, normal distribution) at the harmonic well that is assumed to be the same for all catalytic states. The equilibrium angles of the two catalytic states are set to *n* × 120°−*d*_1_ and *n* × 120°+*d*_2_ with integer *n* (see Fig. 3a,b in main text). For simplicity, we set |*d*_1_|=|*d*_2_|=*d* in the simulation. We estimate the angular increment of the equilibrium angle, Δ*θ*=*d*_2_+*d*_1_=2*d*, to be ∼20° by requiring the ratios of final- and initial-angle MADs obtained from simulating the cases with sharper initial-angle distribution (that is, Scheme 2, Scheme 2 with slow synthesis and Scheme 4 in Fig. 2e,g) match the ratio obtained from experimental data (that is, ∼1.2 shown in Fig. 4f). Note that the ratios of the initial- and final-angle MADs from simulations significantly deviate from the ratios obtained from experiment for 
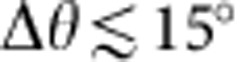
 or 
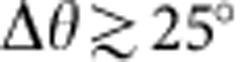
.

Several values of *λ* are used so that the relaxation timescale *λ*^−1^ of the simulation covers the same range of relaxation timescales of *θ* from experiments, that is, 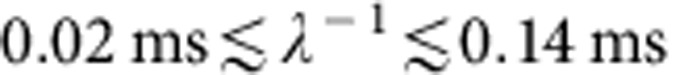 (see Fig. 4g,h). On the other hand, with Δ*θ* chosen to be 20°, the variance *σ*^2^ is set to 220 degree^2^ such that the s.d. of the overall simulated angular distribution of the catalytic dwell (∼17.5°) match those from the experimental angular distributions. Note that only the pause intervals identified by the CP and clustering analyses are used to construct the experimental angular distributions in order to avoid possible artifacts from including the rotation intervals.

On the other hand, the reaction processes are simulated by Monte Carlo techniques as described before^18^. In particular, the angle dependence of the rate constants are adopted from previous experimental studies^17^ as *k*_hyd_=*a*_hyd_ exp(*b*_hyd_*θ*), *k*_Pi_=*a*_Pi_ exp(*b*_Pi_*θ*) and *k*_syn_=*a*_syn_, with *b*_hyd_=0.02 degree^−1^, *b*_Pi_=0.12 degree^−1^ and *θ*=0° corresponding to the angular position of the current catalytic dwell. We set *a*_syn_=*a*_hyd_ for the cases of Schemes 1 and 2 in Fig. 2d,e, and *a*_syn_=0 for the cases of Schemes 1 and 2 with slow synthesis. The remaining constants *a*_hyd_ and *a*_Pi_ are chosen such that the two time constants from the dwell-time distribution of the simulated traces reproduce those fitted from experimental dwell-time distributions with 40 nm bead, that is, purple points in Fig. 4g,h. The numerical values of *a*_hyd_ and *a*_Pi_ used in Fig. 4g,h are given as follows: (1) Scheme 2: *a*_hyd_=1,200 s^−1^=*a*_syn_ and *a*_Pi_=330 s^−1^; (2) Scheme 2 with slow synthesis: *a*_hyd_=890 s^−1^ and *a*_Pi_=330 s^−1^; and (3) Scheme 4: *a*_hyd_=3,600 s^−1^ and *a*_Pi_=300 s^−1^.

In terms of Monte Carlo method, we simulate the transition of the system from one state to another according to the probability of making the corresponding state transition at each simulation step given by *P*(*θ*(*t*))=*k*(*θ*(*t*)) × *δt*, where *θ*(*t*) is the γ-angle at the current simulation time step, *k*(*θ*(*t*)) is the corresponding angle-dependent rate constant for hydrolysis, synthesis or P_i_-release and *δt* is the time interval between simulation steps. Moreover, we simulate long rotary trajectories that contain a large number of catalytic dwells (>10^4^ dwells) to ensure good sampling for the simulated dwell-time statistics.

## Additional information

**How to cite this article:** Li, C.-B. *et al.* ATP hydrolysis assists phosphate release and promotes reaction ordering in F_1_-ATPase. *Nat. Commun.* 6:10223 doi: 10.1038/ncomms10223 (2015).

## Supplementary Material

Supplementary Information Supplementary Figures 1-9, Supplementary Notes 1-4 and Supplementary References.
