# Supplementary material for: ATP hydrolysis assists phosphate release and promotes reaction ordering in F1-ATPase
Source: Nat Commun. 2015 Dec 17;6:10223. doi: 10.1038/ncomms10223 (PMC4703894; doi:10.1038/ncomms10223)
Supplement: Supplementary Information — Supplementary Figures 1-9, Supplementary Notes 1-4 and Supplementary References. [file ncomms10223-s1.pdf]

Supplementary Figures

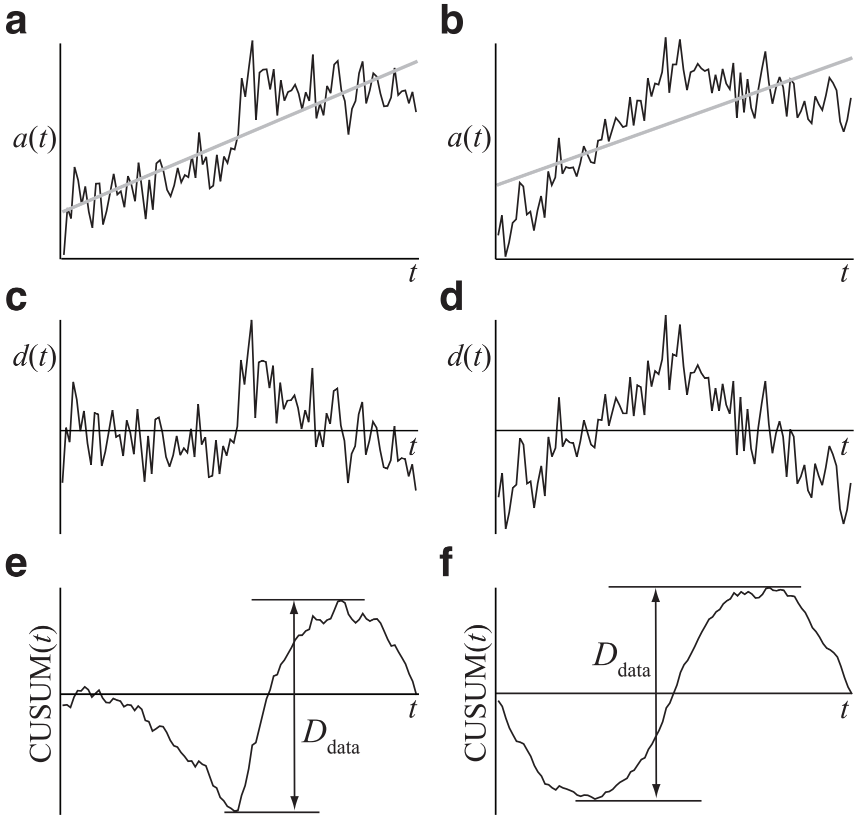

Supplementary Figure 1: Examples of changes in linear trend and CUSUM. (a) The existence of an abrupt jump (a) and a slope change (b). The traces are fitted with straight lines $a_{\rm fit}(t)$ (grey lines). (c) and (d) are the difference time series $d(t) = a(t) - a_{\rm fit}(t)$ of the traces in (a) and (b), respectively. (e) and (f) are the CUSUM curves of (c) and (d), respectively. The total fluctuation of the CUSUM curves are denoted as $D_{\rm data}$.

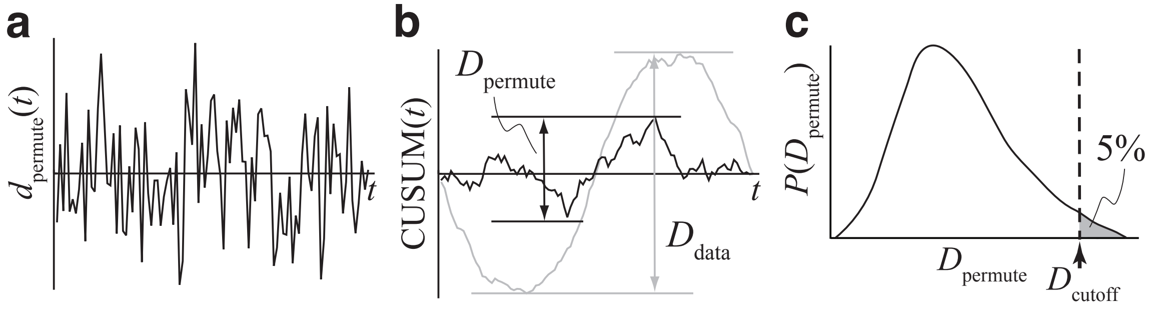

Supplementary Figure 2: Hypothesis test for the existence of change point. (a) A permuted trace obtained by permuting the time ordering of the original one in Supplementary Figure 1d. (b) The CUSUM curve of the permuted trace (dark) with total fluctuation D_{permute} is compared to the original one (grey). (c) The distribution of D_{permute} obtained by generating an ensemble of permuted traces. For a given type I error (e.g., 5%), a cutoff value, D_{cutoff}, can be found such that the null hypothesis (i.e., no change point) is rejected if $D_{\text{data}} \geq D_{\text{cutoff}}$, whereas the null hypothesis is accepted if $D_{\text{data}} < D_{\text{cutoff}}$.

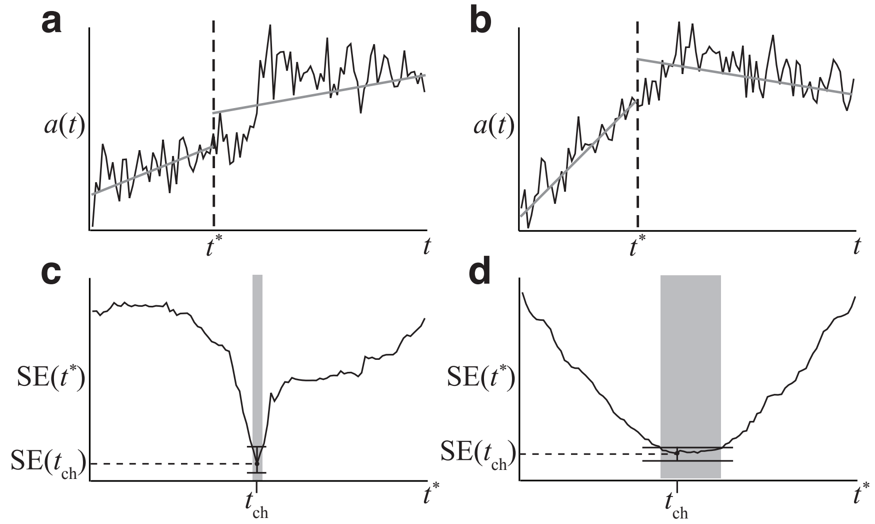

Supplementary Figure 3: Determining change point location and its uncertainty. (a)-(b) Breakdown of the traces into two segments to evaluate the squared error Eq. 2 for the two traces in Supplementary Figures 1a-b. The grey lines are the fitted lines. (c)-(d) The resulting SE(t^*). The change point location t_{ch} is assigned to the time at the global minimum of SE(t^*). The error bar of SE(t_{ch}) at the change point location is estimated by bootstrapping, and the uncertainty in change point location indicated by the grey area is determined as the time interval whose SE values are enclosed by the error bar of SE(t_{ch}).

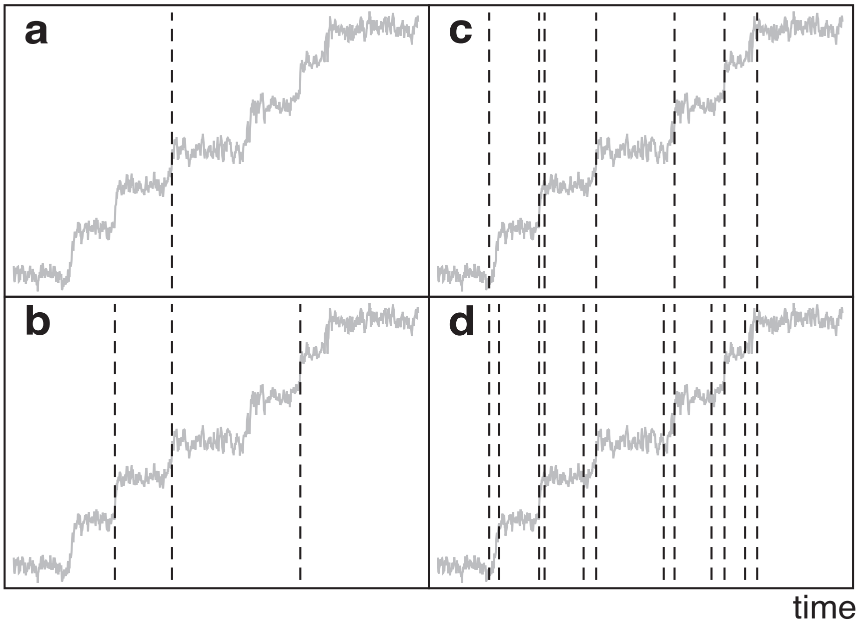

Supplementary Figure 4: Multiple change points are detected by applying the permutation test recursively thereby binary segmenting the time trace. The progression of the detection is from (a) to (d). The dashed lines show the location of the detected change points at each recursive step.

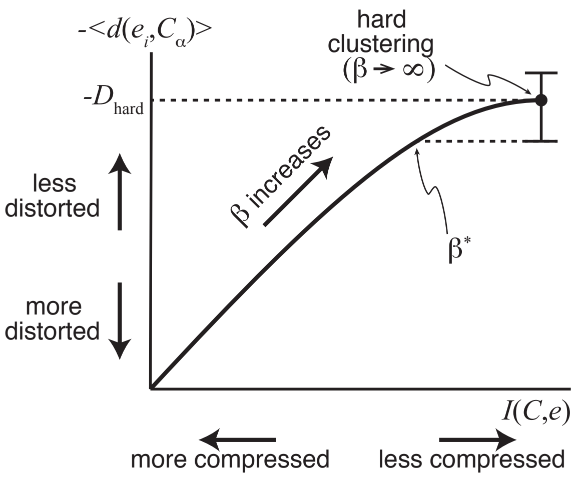

Supplementary Figure 5: Information curve for the tradeoff between compression and distortion with a fixed number of clusters. Error bar represents the sampling and change point location errors in evaluating D_{hard}. β^* is chosen to be the desired "softness" of the clustering to incorporate the effects of error.

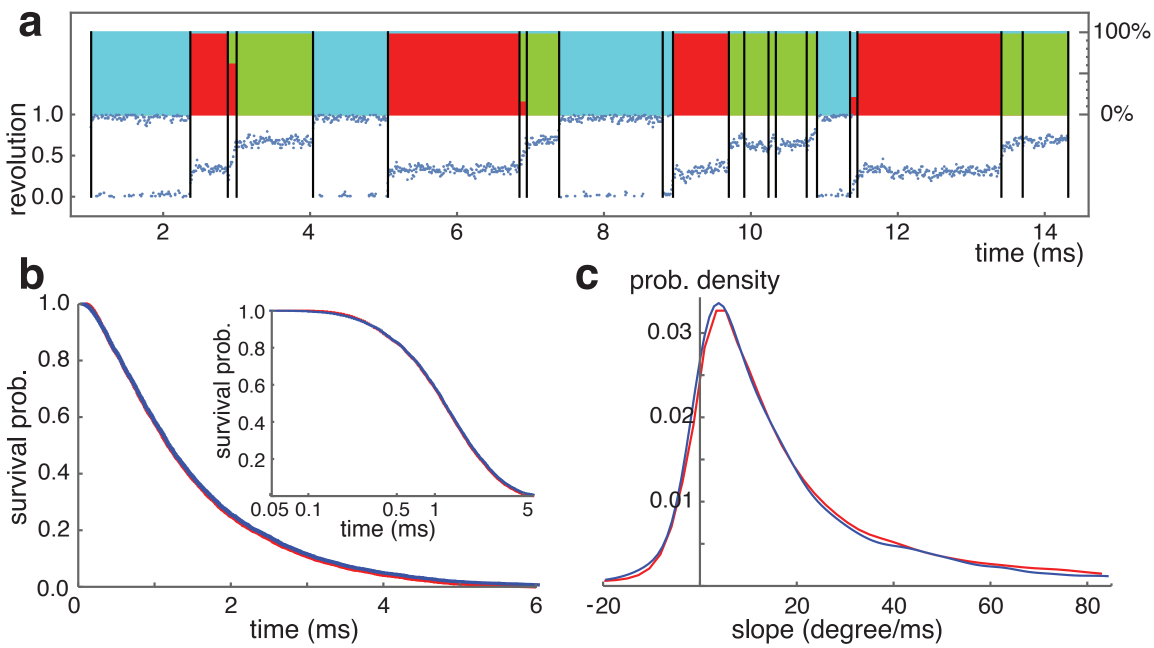

Supplementary Figure 6: Validation of the change point and clustering analyses using rotary trace obtained by simulating the scheme in Figs. 3a-c in the main text. The relaxation time of rotary fluctuations is set to 20 μs, i.e., mimicking the 40 nm bead case (see Figs. 4g-h in the main text). (a) A segment of trace showing the results from change point and clustering analyses. Blue dots: rotary trace. Vertical lines: detected change points. Notations and the rules to assign change point intervals as pause intervals are the same as in Fig. 2a of the main text. Likewise, change points between two pause intervals of the same catalytic dwells are removed. (b) Dwell time survival probability of the pause intervals obtained from the change point and clustering analyses (red line) shows good agreement with the expected dwell distribution of the simulation model (blue line). The expected dwell time of a pause interval of the simulation model corresponds to step 1 to 5 in Figs. 3a-b of the main text. Inset: comparison of the dwell time survival probabilities in linear-log scale to show agreements at the short timescales. (c) Agreement of slope distribution obtained from the change point and clustering analyses (red line) with the expected distribution of the simulation model (blue line). The expected slope distribution of the simulation model is obtained by fitting the simulated rotary trace from step 1 to 5 in Figs. 3a-b of the main text by a straight line. The validations in (b) and (c) show that the change point and clustering analyses can reliably extract the dwell time statistics.

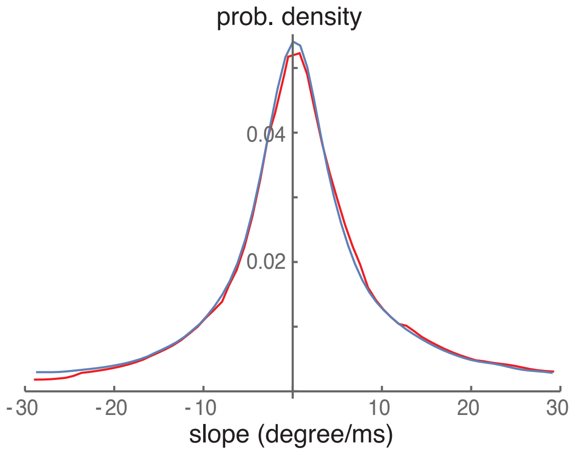

Supplementary Figure 7: Unbiased slope distribution for the case when there is no angular increment between the pre- and post-hydrolysis states in Figs. 3a-b of the main text. i.e., $d_1 = d_2 = 0°$. Red line: distribution obtained from change point and clustering analyses of the simulated rotary trace. Blue line: expected distribution from the simulation model. The rotary trace is obtained by simulating the scheme in Figs. 3a-c of the main text, with the relaxation time of rotary fluctuations set to 20 μs, i.e., mimicking the 40 nm bead case. This validation shows that the change point and clustering analyses reliably extract the unbiased slope distribution when there is no angular increment at the catalytic dwells.

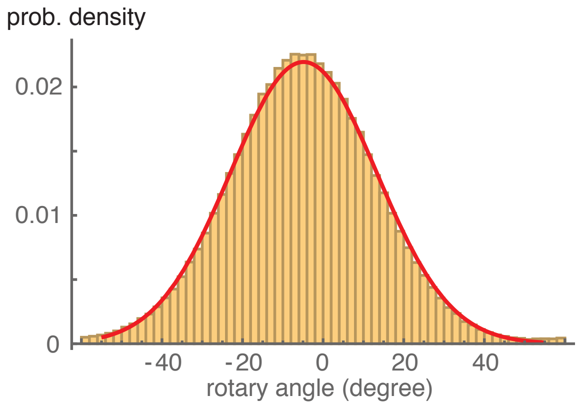

Supplementary Figure 8: Demonstration of the difficulty to detect the small angular increment from the angular histogram of the catalytic dwell. The histogram is obtained from the rotary trace by simulating the scheme in Figs. 3a-c in the main text, with relaxation time of rotary fluctuations set to 20 μs, i.e., mimicking the 40 nm bead case, and with angular increment between pre- and post-hydrolysis states set to 20°. Red line: fitting of the rotary angles with a normal distribution. The deviation of the histogram from the fitted normal distribution is insignificant to tell that there is an angular increment at the catalytic dwell.

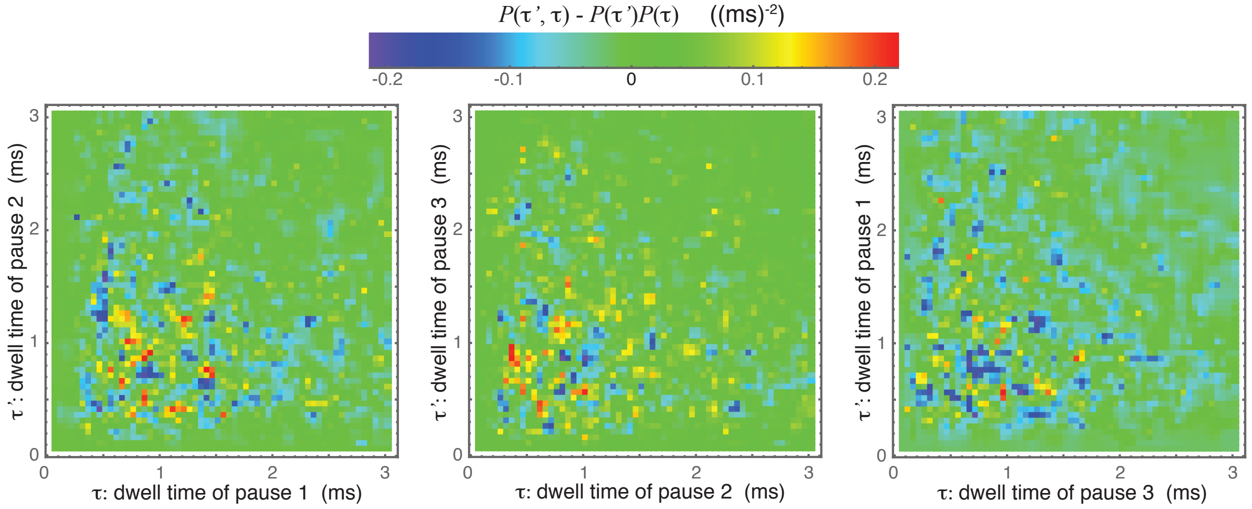

Supplementary Figure 9: 2D correlograms for $P(\tau', \tau) - P(\tau')P(\tau)$, with the dwell time of the current catalytic dwell τ and the next catalytic dwell τ' extracted from the single F_1 experimental data by change point and clustering analyses. $P(\tau', \tau)$: joint probability density of τ' and τ. $P(\tau')$ (and $P(\tau)$): probability density of τ' (and τ). The correlograms from the three catalytic dwells, i.e., pause 1 to 2, pause 2 to 3, and pause 3 to 1, are plotted separately from the left to right, respectively. $(P(\tau', \tau) - P(\tau')P(\tau))$ has small values fluctuating randomly around zero indicating that τ' and τ are not correlated. The lack of correlation between τ' and τ is further confirmed by permutation test for the Pearson correlation coefficient (PCC) of τ' and τ as follows: Let $\{(\tau_1', \tau_1), (\tau_2', \tau_2), \cdots\}$ be the set of consecutive dwell times obtained from the experimental trace and C_{data} be the PCC of this set. We permute the positions of τ_i in the set $\{(\tau_1', \tau_1), (\tau_2', \tau_2), \cdots\}$ to remove the correlation (if there is any) between τ' and τ, and compute the permuted PCC. This permutation procedure is repeated many times and a set of permuted PCCs is generated. The original C_{data} is then compared to the set of permuted PCCs to obtain the two-sided p-value. For all experimental traces considered in this work, the two-sided p-values are always larger than, e.g., 5%, indicating that no correlation between τ' and τ can be detected with statistical significance.

Supplementary Notes

Supplementary Note 1: Testing the existence of change points by permutation method

We generalize the change point algorithm developed by Taylor [1], which detects changes in the mean values, to detect both sudden jumps and changes in the linear trend (see e.g., Supplementary Figures 1a and b) in a time series. To decide if change points exist in a time series, permutation test is employed to test the two hypotheses: There is no change point (the null hypothesis), and there exists at least one change point (the alternative hypothesis). Here we first describe how to detect the existence of a single change point, and the detection of multiple change points will be discussed in Supplementary Note 3.

Suppose we have a segment of time series as shown in Supplementary Figure 1a or b, we decide if change point(s) exists by comparing the statistical significance of the null and alternative hypotheses in terms of the cumulative sum (CUSUM) of the time series as follows: The time series $a(t)$ is first fitted by a straight line $a_{\text{fit}}(t)$ (Supplementary Figures 1a-b), and the difference time series, $d(t) = a(t) - a_{\text{fit}}(t)$, is constructed (Supplementary Figures 1c-d). The CUSUM is defined by the cumulative sum of the difference time series as

$$\text{CUSUM}(t) = \sum_{t'=1}^{t}(a(t') - a_{\text{fit}}(t')). \tag{1}$$

The CUSUMs for the two traces in Supplementary Figures 1a-b are shown in Supplementary Figures 1e-f. It is expected that the total fluctuation of CUSUM curve, denoted by $D_{\text{data}} = \max(\text{CUSUM}(t)) - \min(\text{CUSUM}(t))$, has a bigger value if it is more likely that a change point exists, i.e., the fitted straight line $a_{\text{fit}}(t)$ cannot describe well the trends in the time series $a(t)$. In order to justify how big the value of D_{data} is enough to reject the null hypothesis, we need to estimate the range of values D_{data} can take when no change point exists.

To create such ensemble of traces with no change points, we permute the time ordering of the difference time series $d(t)$ to obtain the permuted traces $d_{\text{permute}}(t)$. Supplementary Figure 2a shows an example of $d_{\text{permute}}(t)$ obtained by permuting the trace $d(t)$ in Supplementary Figure 1d. It is expected that the random permutation should wash away any change point in the original trace $d(t)$. The CUSUM of the permuted trace $d_{\text{permute}}(t)$ is again evaluated and the total fluctuation D_{permute} can be obtained (see Supplementary Figure 2b). Similarly, an ensemble of permutation traces is generated to create the distribution

of D_{permute} as shown in Supplementary Figure 2c. The distribution $P(D_{\text{permute}})$ provides an estimation of how big the total fluctuation in the CUSUM can be when no change point exists.

We declare the existence of change point(s) when the null hypothesis can be rejected with more than a given confidence, e.g., 95%, by determining the cutoff value D_{cutoff} (see Supplementary Figure 2c) such that the probability of finding $D_{\text{permute}} \geq D_{\text{cutoff}}$ (shaded area in Supplementary Figure 2c) equals to $(100 - 95)\% = 5\%$. If we have $D_{\text{data}} \geq D_{\text{cutoff}}$ from the original time series, the null hypothesis can be rejected with confidence $\geq 95\%$ and we declare the existence of at least one change point, whereas no change point is concluded if $D_{\text{data}} < D_{\text{cutoff}}$. The 95%-confidence used above to reject the null hypothesis also represents a 5% probability of type I error, where the type I error denotes the probability to have a false positive, i.e., the chance for the algorithm to conclude that a change point exists even it really does not. One can in general choose the value of type I error in a change point detection. A smaller type I error corresponds to a more conservative assignment of change point (i.e., with a larger value of D_{cutoff}) and therefore results in a smaller number of detected change points.

On the other hand, the type II error, that corresponds to the probability of missing change point at time instant where change point actually exists, is not explicitly controlled in our algorithm. To avoid the missing a large number of change points in the detection, the choice of an extremely small value of type I error should be avoided, i.e., the assignment of change point should not be too conservative. Some undesired change points resulted from the detection can later be removed by the clustering procedure discussed below in Supplementary Note 4.

Supplementary Note 2: Determining the location of change point and its uncertainty

If the null hypothesis (i.e., no change point exists) is rejected, we declare that change point(s) exists and the next step is to determine the location of the most prominent change point. We use the squared error (SE) to determine the location. Supplementary Figures 3a-b demonstrate the detection using the traces in Supplementary Figures 1a-b. For a given

time t^* $(1 < t^* < T)$, the SE is defined as

$$\mathrm{SE}(t^*) = \sum_{t'=1}^{t^*}(a(t') - a_{\mathrm{fit}}^{\mathrm{L}}(t'))^2 + \sum_{t'=t^*+1}^{T}(a(t') - a_{\mathrm{fit}}^{\mathrm{R}}(t'))^2, \quad (2)$$

where T is the number of data points, $a_{\mathrm{fit}}^{\mathrm{L}}(t')$ and $a_{\mathrm{fit}}^{\mathrm{R}}(t')$ are, respectively, straight lines fitted to the left and right segments of the time series separated at $t = t^*$ (grey lines in Supplementary Figures 3a-b). The resulting $\mathrm{SE}(t^*)$ are shown in Supplementary Figures 3c-d. The time t_{ch} at which $\mathrm{SE}(t^*)$ reaches its minimum represents the location where the left and right segments are best described by the fitted straight lines, and therefore serves as the best estimation of the change point location. If there exist multiple change points in the time series, $\mathrm{SE}(t^*)$ can have several minimum and the global minimum is chosen as the location of the most prominent change point.

Next we provide a simple scheme based on bootstrapping method [2] to estimate the error bar associated with the determined change point location. The error bar represents the uncertainty in pinpointing the change point location due to the sampling error in evaluating the SE. Suppose that the change point location is determined to be at $t^* = t_{\mathrm{ch}}$, we first estimate the uncertainty in $\mathrm{SE}(t_{\mathrm{ch}}) = \sum_{t'=1}^{t^{\mathrm{ch}}}(a(t') - a_{\mathrm{fit}}^{\mathrm{L}}(t'))^2 + \sum_{t'=t^{\mathrm{ch}}+1}^{T}(a(t') - a_{\mathrm{fit}}^{\mathrm{R}}(t'))^2$ by bootstrapping method as follows: The segment of data, $\{a(1), \cdots, a(t_{\mathrm{ch}})\}$, under the first summation in evaluating $\mathrm{SE}(t_{\mathrm{ch}})$ is resampled with replacement (i.e., bootstrapping). Similarly a bootstrap resampling is performed for the second segment $\{a(t_{\mathrm{ch}} + 1), \cdots, a(T)\}$. As before, we fit each of these two bootstrapped segments with straight line, and the bootstrapped squared error $\mathrm{SE}_1^{\mathrm{boot}}(t_{\mathrm{ch}})$ is evaluated. This process is repeated many times (usually ~ 1000 times) to generate an ensemble of bootstrapped SE, i.e., $\{\mathrm{SE}_1^{\mathrm{boot}}(t_{\mathrm{ch}}), \cdots, \mathrm{SE}_{1000}^{\mathrm{boot}}(t_{\mathrm{ch}})\}$, which gives the bootstrap distribution of $\mathrm{SE}(t_{\mathrm{ch}})$. The error bar (shown in Supplementary Figures 3c-d) associated with the value of $\mathrm{SE}(t_{\mathrm{ch}})$ corresponds to the bootstrap 68% confidence interval, i.e., the interval from the 16th percentile to the 84th percentile of the bootstrap distribution. Since any time instant t^* in the neighborhood of t_{ch} can potentially be a change point if the value of $\mathrm{SE}(t^*)$ falls inside the error bar of $\mathrm{SE}(t_{\mathrm{ch}})$, we simply denote the uncertainty in the change point location (grey area in Supplementary Figures 3c-d) by the time interval whose SE values are enclosed by the error bar of $\mathrm{SE}(t_{\mathrm{ch}})$.

It can be easily seen that the size of the error bar in the change point location depends on the number of data points before and after a change point, and on how prominent a change point is. For instance, the presence of a larger number of data points before and after a

change point can give rise to a smaller error bar for the value of $\mathrm{SE}(t_{\mathrm{ch}})$, and therefore results in a smaller uncertainty in the change point location. Likewise, a prominent change point, e.g., a big jump or slope change, results in a steep $\mathrm{SE}(t^*)$ curve with a sharp minimum (see e.g., Supplementary Figure 3c) which can also lead to a smaller uncertainty in the estimated change point location.

Supplementary Note 3: Detecting multiple change points recursively

To detect and locate multiple change points in the time trace, the algorithm above is applied recursively by binary segmentation. Supplementary Figure 4 shows the progression in identifying multiple change points at different recursive steps. For a given segment of time series containing multiple change points, we first apply the algorithm discussed above to decide if change point(s) exists and to locate the most prominent change point (Supplementary Figure 4a). The trace is then divided into two disjoint segments separated by the change point just found. The permutation test is applied again to each of the segments to identify additional change points (see Supplementary Figure 4b). The binary segmentation is then repeated (Supplementary Figures 4c-d) until no change points could be found in the segments anymore.

Finally, we note that the resulting change points from the above procedure of binary segmentation may contain some error both in the hypothesis tests for their existence and in their location estimations. This is because the change points on the left and right hand sides of the segmentation, that will be detected in later stages of the binary segmentation (e.g. the change points that have not yet been detected in Supplementary Figure 4a), can defect the hypothesis test and the estimation of change point location at the current stage of the segmentation. Therefore, we perform a final clean-up procedure as follows: Let the locations of the multiple change points resulted from the binary segmentation be t_i for the i-th change point with $i = 1, 2, 3, \cdots$ and $t_1 < t_2 < t_3 < \cdots$. The hypothesis test and location estimation are carried out again for each change point at t_i by only using the segment of the time series from t_{i-1} to t_{i+1}. In this way, the existence and location of each change point can be evaluated more precisely free from the effect of the undetected change points.

Supplementary Note 4: Clustering to assign change point intervals to catalytic dwells

We first introduce the concept of "soft" clustering as follows. Given N_e elements,

$\{e_1, e_2, \cdots, e_{N_e}\}$, "hard" clustering algorithm assigns each element to exactly one cluster (or group) out of N_c clusters, $\{C_1, C_2, \cdots, C_{N_c}\}$ with $N_c \leq N_e$. On the other hand, soft clustering allows the elements to belong to more than one cluster with a certain "membership". These memberships are specified by the conditional probability $P(C_\alpha|e_i)$ (with $\sum_{\alpha=1}^{N_c} P(C_\alpha|e_i) = 1$) for the given element e_i belonging to the cluster C_α. The hard clustering is a special case of soft clustering in which $P(C_\alpha|e_i)$ equals to either zero or one.

Clustering procedures assign dissimilar elements to distinct clusters and so one needs to provide a distance (or dissimilarity measure), $d(e_i, e_j)$ (with $d(e_i, e_j) = d(e_j, e_i)$ and $d(e_i, e_i) = 0$), between the elements. In our study of the rotary time series of F_1-ATPase, the distance between two change point intervals (the elements) is chosen to be the difference between the mean angles of the intervals. From the element-to-element distance $d(e_i, e_j)$, one can obtain the element-to-cluster distance $d(e_i, C_\alpha)$ as the weighted average,

$$d(e_i, C_\alpha) = \sum_{j=1}^{N_e} P(e_j|C_\alpha) d(e_i, e_j), \tag{3}$$

where $P(e_i|C_\alpha) = P(C_\alpha|e_i)P(e_i)/P(C_\alpha)$ is the probability of finding the element e_i in the cluster C_α. The distortion in the cluster description represents the averaged element-to-cluster distance,

$$\begin{aligned} \langle d(e_i, C_\alpha)\rangle_{P(e_i,C_\alpha)} &= \sum_{i=1}^{N_e}\sum_{\alpha=1}^{N_c} P(e_i, C_\alpha) d(e_i, C_\alpha) \\ &= \sum_{\alpha=1}^{N_c} P(C_\alpha)\left[\sum_{i,j=1}^{N_e} P(e_i|C_\alpha)P(e_j|C_\alpha)d(e_i, e_j)\right]. \end{aligned} \tag{4}$$

The second line of Eq. 4 tells us that the distortion is the intra-cluster distance (the term inside the square brackets) averaged over the clusters. It can be easily checked that $0 \leq \langle d(e_i, C_\alpha)\rangle_{P(e_i,C_\alpha)} \leq \sum_{i,j=1}^{N_e} P(e_i)P(e_j)d(e_i, e_j)$. The distortion obtains its minimum value at zero when there are $N_c = N_e$ clusters and each element is itself a cluster, i.e., no compression. In this case, we simply have $P(C_\alpha|e_i) = P(e_i|C_\alpha) = \delta_{\alpha,i}$ ($\delta_{\alpha,i} = 1$ if $\alpha = i$ and $\delta_{\alpha,i} = 0$ otherwise) and $P(C_\alpha) = P(e_i)$. The maximum distortion is reached when there is only one cluster ($N_c = 1$ and $P(C_1) = 1$) and all elements are assigned to this cluster, i.e., $P(C_1|e_i) = 1$ for $i = 1, \cdots, N_e$. This corresponds to the maximally compressed case.

On the other hand, the mutual information [3] between the elements and the clusters,

$$I(C, e) = \sum_{i=1}^{N_e}\sum_{\alpha=1}^{N_c} P(C_\alpha, e_i)\log\left[\frac{P(C_\alpha|e_i)}{P(C_\alpha)}\right] \geq 0, \tag{5}$$

provides a measure to quantify the degree of compression (or clustering) described by the membership $P(C_\alpha|e_i)$ with N_c clusters. Here $P(C_\alpha, e_i) = P(C_\alpha|e_i)P(e_i)$. In the least compressed case when there are $N_c = N_e$ clusters (i.e., each element is itself a cluster), $I(C, e) = H(e) = -\sum_{i=1}^{N_e} P(e_i) \log P(e_i)$, which is just the information content of the elements. In the maximum compressed case when there is only one cluster ($N_c = 1$) (i.e., all elements are assigned to a single cluster), $I(C, e) = 0$ and the cluster carries no information of the elements.

Rate distortion theory [3-5], developed by Claude Shannon in his foundational work on information theory, formulates the tradeoff between compression and distortion to find the most compressed description of the elements for a given degree of distortion. Suppose there are N_c clusters, the tradeoff corresponds to minimize the mutual information $I(C, e)$ with respect to $P(C_\alpha|e_i)$ subject to the constraint $\langle d(e_i, C_\alpha)\rangle_{P(e_i,C_\alpha)} = \mathcal{D}$, where $\mathcal{D}$ is the desired value for the distortion. The solution to this constrained optimization problem can be obtained using the method of Lagrange multiplier, where we minimize the Lagrange function,

$$L = I(C, e) + \beta\langle d(e_i, C_\alpha)\rangle, \tag{6}$$

with respect to $P(C_\alpha|e_i)$ with the Lagrange multiplier $\beta \geq 0$. The formal expression of $P(C_\alpha|e_i)$ that minimizes Eq. 6 (i.e., by setting $\partial L/\partial P(C_\alpha|e_i) = 0$) is given by [3],

$$P(C_\alpha|e_i) = \frac{P(C_\alpha) \exp\left[-\beta d(e_i, C_\alpha)\right]}{Z(e_i, \beta)}, \tag{7}$$

where $Z(e_i, \beta)$ is the "generalized" partition function,

$$Z(e_i, \beta) = \sum_{\alpha'=1}^{N_c} P(C_{\alpha'}) \exp\left[-\beta d(e_i, C_{\alpha'})\right], \tag{8}$$

to ensure correct normalization (i.e., $\sum_{\alpha'=1}^{N_c} P(C_{\alpha'}|e_i) = 1$).

The expression Eq. 7 only serves as a formal solution since the right hand side of the equality also depends on $P(C_\alpha|e_i)$ through

$$P(C_\alpha) = \sum_{i=1}^{N_e} P(C_\alpha|e_i)P(e_i) \tag{9}$$

and

$$P(e_i|C_\alpha) = \frac{P(C_\alpha|e_i)P(e_i)}{\sum_{j=1}^{N_e} P(C_\alpha|e_j)P(e_j)}. \tag{10}$$

Moreover, the actual value of the Lagrange multiplier β have to be determined by requiring $\langle d(e_i, C_\alpha)\rangle_{P(e_i,C_\alpha)} = \mathcal{D}$ where $P(C_\alpha|e_i)$ has the form of Eq. 7. In practice, the determination of $P(C_\alpha|e_i)$ in the optimization problem is solved numerically by an iterative procedure, called the Blahut-Arimoto algorithm [3], in terms of the self-consistent equations Eq. 7, 9 and 10. For given N_c ($N_c = 3$ in the current study for the 3 catalytic dwells) and β, the iterative procedure is as follows:

[Step 1] $P(C_\alpha|e_i)$ ($\alpha = 1, \cdots, N_c$, $i = 1, \cdots, N_e$) are randomly generated with $\sum_{\alpha=1}^{N_c} P(C_\alpha|e_i) = 1$.

[Step 2] $P(C_\alpha)$ and $P(e_i|C_\alpha)$ are evaluated using Eq. 9 and Eq. 10, respectively. The element-to-cluster distance $d(e_i, C_\alpha)$ are then evaluated by Eq. 3. Next the partition functions $Z(e_i, \beta)$ can be obtained using Eq. 8.

[Step 3] The memberships are then updated from $P(C_\alpha|e_i)$ to $P'(C_\alpha|e_i)$ using Eq. 7 as $P'(C_\alpha|e_i) = P(C_\alpha)\exp\left[-\beta d(e_i, C_\alpha)\right]/Z(e_i, \beta)$.

[Step 4] The procedure stops if the updated memberships, $P'(C_\alpha|e_i)$, agree with the old one, $P(C_\alpha|e_i)$, up to a chosen precision. If convergence is not reached yet, Step 2 to 4 are repeated with the initial memberships replaced by the updated one, i.e., setting $P(C_\alpha|e_i) = P'(C_\alpha|e_i)$.

We note that generally the above procedure may not lead to the global minimum of the Lagrange function and therefore multiple runs (> 20 in this work) with different initial conditions, i.e., with different set of $P(C_\alpha|e_i)$ in Step 1 above, are performed and the iteration result with the minimum value of the Lagrange function is used.

Before addressing how we fix the value of β that is required as an input to the iterative procedures for a given number of cluster N_c, we first give some intuitions on the meaning of β and its relation to the "softness" of the clustering. First let us consider the case when β is a very large positive number. For a given element e_i, the partition function in Eq. 8, $Z(e_i, \beta) = \sum_{\alpha'=1}^{N_c} P(C_{\alpha'})\exp\left[-\beta d(e_i, C_{\alpha'})\right] \xrightarrow[\beta\ \text{large}]{} P(C_{\alpha''})\exp[-\beta d(e_i, C_{\alpha''})]$, where $C_{\alpha''}$ is the cluster having the smallest element-to-cluster distance with e_i, i.e., $d(e_i, C_{\alpha''})$ is the smallest among all N_c clusters. After substituting $Z(e_i, \beta) \to P(C_{\alpha''})\exp[-\beta d(e_i, C_{\alpha''})]$ into Eq. 7, one obtains $P(C_\alpha|e_i) = \delta_{\alpha,\alpha''}$ as $\beta \to \infty$. Therefore, the large β case corresponds

to the hard clustering case in which the element e_i is assigned to the cluster $C_{\alpha''}$ having the smallest element-to-cluster distance with e_i. Moreover, one can see in this case that the Lagrange function (Eq. 6) is dominated by the second term, $\beta\langle d(e_i, C_\alpha)\rangle$, and so the minimization problem reduces to the minimization of the distortion only.

On the other hand, $P(C_\alpha|e_i)$ (Eq. 7) becomes independent of C_α when $\beta = 0$. This means that each element is equally assigned to the clusters, i.e., the softest clustering case. In this case even for $N_c > 1$, there is no need to distinguish the clusters as their membership $P(C_\alpha|e_i)$ are the same, so there exists effectively only one cluster and all elements belong to it, i.e., the maximally compressed case. This can also be seen from the Lagrange function (Eq. 6) that when $\beta = 0$, the minimization reduces to the minimization of the compression (the mutual information) only. In general, the Lagrange multiplier β characterizes both the degree of softness, and the tradeoff between compression and distortion in the clustering. Such tradeoff can be visualized by the information curve as shown in Supplementary Figure 5 in which each point on the curve represents the best compression (i.e., with the lowest mutual information) given the corresponding distortion value.

We now move on to the determination of the degree of softness. Here we adopt an error-based algorithm [6] to determine the appropriate softness (i.e., the value of β) for the clustering as follows. We first perform hard clustering of the elements with a large β corresponding to the black dot in the information curve in Supplementary Figure 5 and let us denote the corresponding value of the distortion as $D_{\text{hard}} = \langle d(e_i, C_\alpha)\rangle|_{\text{hard}}$. One can think that the obtained hard clustering corresponds to the case when there is no error in evaluating the element-to-element distance $d(e_i, e_j)$ so that the change point intervals can be assigned to a particular cluster unambiguously. However, in practice there exist several errors that can affect the evaluation of $d(e_i, e_j)$, which include the uncertainty in the change point locations that affects the range of data points belonging to each change point interval, and the sampling error to evaluate the mean angle of the change point intervals with finite number of data points. Therefore, the assignment of the elements, especially for those located near the cluster boundaries, to the clusters can be fuzzy (i.e., soft).

To determine the degree of softness originated from the errors in evaluating $d(e_i, e_j)$, we estimate the error in D_{hard} associated with the sampling error and uncertainties in change point location in terms of the bootstrapping method [2] similar to those in the change point detection. The idea is to evaluate the bootstrapped mean angle by resampling with replacement

the data points inside a change point interval whose boundaries are randomly chosen according to the error bars of the change point location. Using the bootstrapped mean angles of all the change point intervals, the bootstrapped distances $d^{\text{boot}}(e_i, e_j)$ $(i, j = 1, \cdots, N_e)$ can be calculated and the hard clustering procedure is performed to obtain the bootstrapped distortion $D^{\text{boot}}_{\text{hard}}$. This bootstrapping procedure is then repeated for many times (usually 1000 times) to generate an ensemble of bootstrapped distortion, $\{D^{\text{boot}}_{\text{hard},1}, D^{\text{boot}}_{\text{hard},2}, \cdots, D^{\text{boot}}_{\text{hard},1000}\}$, which gives the bootstrap distribution of $D^{\text{boot}}_{\text{hard}}$ representing the possible variations of D_{hard} due to the sampling and change point location errors. The error bar (shown in Supplementary Figure 5) associated with the value of D_{hard} corresponds to the bootstrap 68% confidence interval, i.e., the interval from the 16th percentile to the 84th percentile of the bootstrap distribution. Let β^* be the largest value of β that falls outside the error bar of D_{hard} (Supplementary Figure 5), any $\beta > \beta^*$ then represents clustering with too small distortion that can be allowed by the sampling and change point location errors. Therefore, we choose β^* as the desired value of β which also fixes the degree of softness in the clustering. One can easily see that β^* is smaller and the clustering is softer if the sampling and change point errors are bigger, simply reflecting the fact that the assignment of the elements to the clusters become more ambiguous.

Supplementary References

[1] Taylor, W. A. (2000). Change-point analysis: A powerful new tool for detecting changes, available at http://www.variation.com/cpa/tech/changepoint.html.

[2] Efron, B. Nonparametric Estimates of Standard Error: The Jackknife, the Bootstrap and Other Methods. *Biometrika* **68**, 589–599 (1981).

[3] Cover, T. M. & Thomas, J. A. *Elements of Information Theory* (John Wiley & Sons, Inc., 1991).

[4] Shannon, C. E. A mathematical theory of communication. *Bell Sys. Tech. J.* **27**, 379–423 (1948).

[5] Shannon, C. E. A mathematical theory of communication. *Bell Sys. Tech. J.* **27**, 623–656 (1948).

[6] Taylor, J. N., Li, C.-B., Cooper, D. R., Landes, C. F. & Komatsuzaki, T. Error-based extraction of states and energy Landscapes from experimental single-molecule time-series. *Sci. Rep.* **5**, 9174 (2015).
